# Supplementary material for: Bud-Poplar-Extract-Embedded Chitosan Films as Multifunctional Wound Healing Dressing
Source: Molecules. 2022 Nov 10;27(22):7757. doi: 10.3390/molecules27227757 (PMC9695786; doi:10.3390/molecules27227757)
Supplement: Supplementary file 1 [file molecules-27-07757-s001.zip › molecules-1924125-supplementary.pdf]

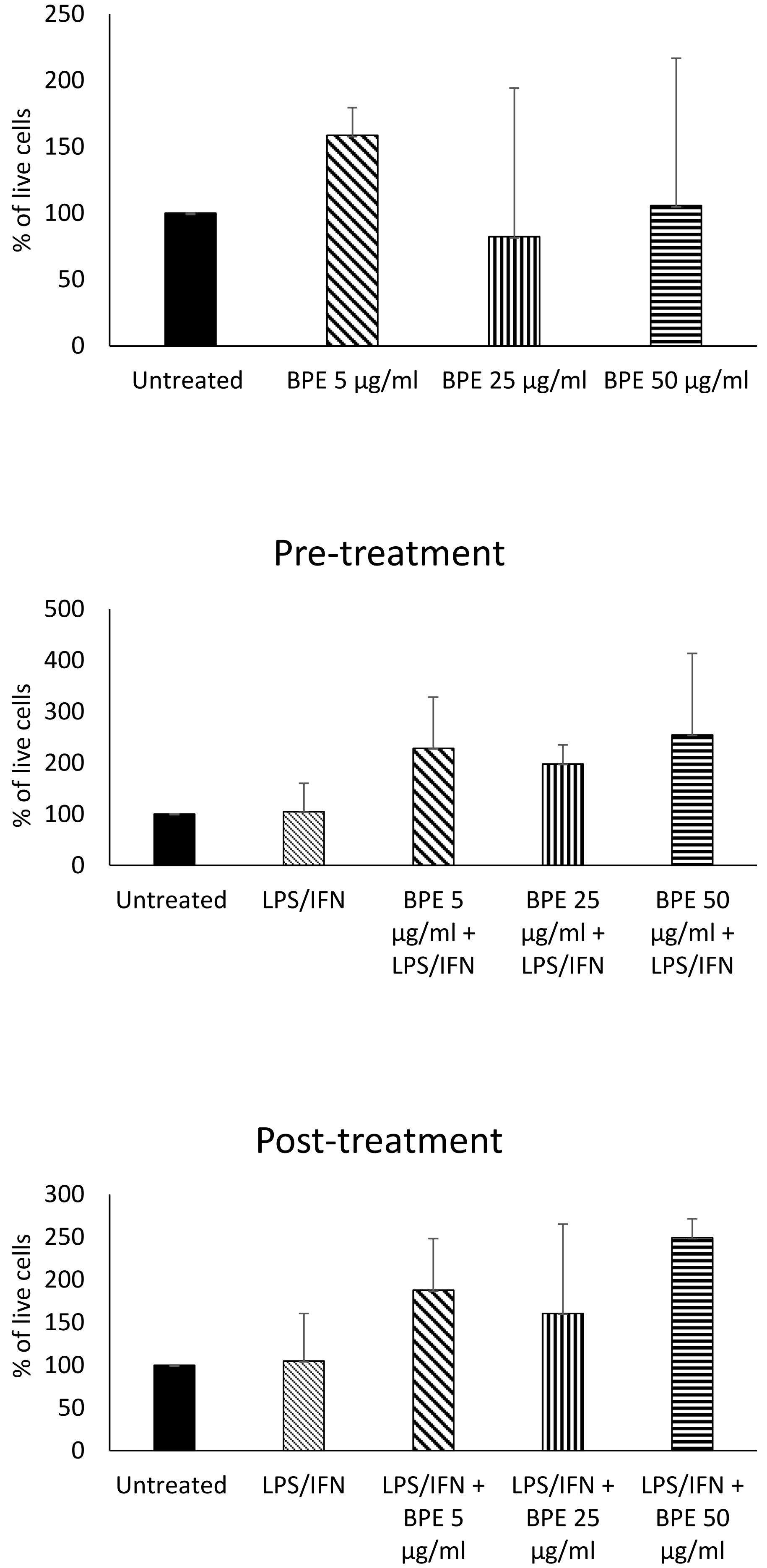

Figure S1. Citotoxicity of BPE on human monocyte-macrophages. Results are expressed as percentage of live cells with respect to untreated cells, considered 100. The results are expressed as mean ± SD of three different measures.
